# *Acinetobacter Baumannii* isolates: epidemiology, antibiogram and nosocomial status studied over a 25 month period in a tertiary care hospital in India

**DOI:** 10.1186/1753-6561-5-S6-P291

**Published:** 2011-06-29

**Authors:** N Jaggi, P Sissodia, L Sharma

**Affiliations:** 1 Labs & Infection Control, Artemis Health Institute, Gurgaon, Haryana, India

## Introduction / objectives

The emergence of *Acinetobacter baumannii* as an epidemiologically significant nosocomial agent based on its epidemiology, antibiogram patterns and clinical correlation was explored in a 25 month study at a tertiary care hospital in India.

## Methods

The *Acinetobacter baumannii* isolates over a twenty-five month period (Dec’08-Dec’10) were studied retrospectively for their antibiotic patterns, pathogenic stautus and epidemiology with special reference to nosocomial acquisition.

## Setting

Superspeciality Tertiary care Indian hospital.

## Results

*A. baumannii* were isolated in 354 samples out of 3036 gram negative isolates (11.6% prevalence) from the entire hospital in the 25 month period. Maximum isolates were from respiratory secretions (59.8%) followed by blood (18.6%). Prevalence of *A. baumannii* rose to 29.5% (269 out of 909 gram negatives isolates) in ICU. The nosocomial status of *A. baumannii* was revealed in its contribution to 39.3% VAP, 38.7% CA-BSI, 12.6% SSI and 16.9% CA-UTI. Overall resistance of *A. baumannii* for carbapenems was 89% from all hospital isolates. ICU isolates showed higher resistance (92.9%) as compared to IPD (83.8%) and OPD (47.0%).

## Conclusion

*A. baumannii* is mainly an ICU bug, showing 75.9% prevalence (269 isolates out of 354). Overuse of carbapenems in the ICU setting probably led to selection pressure and high level resistance of Acinetobacter to them. Hence implementation of antibiotic policy for judicious use of antibiotics should be stressed on. Also, one must prevent the nosocomial spread of Acinetobacter by appropriate infection control measures.

## Disclosure of interest

None declared.

